# A Neurological Manifestation of Anaplasmosis: A Case Report

**DOI:** 10.7759/cureus.77877

**Published:** 2025-01-23

**Authors:** Siddartha Guru, Marvi Mahar, Navami Guru, Leslie Parent

**Affiliations:** 1 Infectious Diseases, Penn State Health Milton S. Hershey Medical Center, Hershey, USA; 2 Infectious Diseases, Case Western Reserve University/University Hospitals Cleveland Medical Center, Cleveland, USA; 3 Internal Medicine, Greater Baltimore Medical Center, Towson, USA

**Keywords:** anaplasma phagocytophilum, neurological manifestations, rare case report, stroke, tick-borne infections

## Abstract

Human granulocytic anaplasmosis (HGA) is a tick-borne infection caused by* *a small intracellular gram-negative bacteria called* Anaplasma phagocytophilum. *It is a multisystemic disease, but neurological manifestations are rare.* *We report a rare neurological manifestation of stroke in a 65-year-old woman who presented initially with abdominal pain, nausea, vomiting, and dizziness. She developed worsening renal function and encephalopathy requiring hemodialysis and intubation for airway protection. Brain imaging showed acute infarcts. Morulae were seen on the peripheral smear. The diagnosis was confirmed with a positive *Anaplasma* PCR. Her mentation improved after 48 hours on doxycycline.

## Introduction

Human granulocytic anaplasmosis (HGA) is a tick-borne infection caused by a small intracellular gram-negative bacteria called *Anaplasma phagocytophilum *and transmitted by the *Ixodes scapularis *tick. Anaplasmosis has been on the rise over the past decade in the US. Per the Centers for Disease Control and Prevention (CDC), 348 cases were reported in 2000 compared to 5,651 cases in 2022 [1]. The majority of the cases are reported in the northeast region of the country, including Pennsylvania. Per the Pennsylvania Department of Health, 1292 cases were reported in 2023 alone [2]. Climate change, improved awareness from clinicians, and an increase in human interactions with tick and deer habitats have all contributed to the rising cases [3]. The most common symptoms include fevers, chills, headaches, myalgias, arthralgias, abdominal pain, nausea, vomiting, and diarrhea [3-5]. Complications include acute renal failure, respiratory failure, disseminated intravascular coagulation (DIC), hemophagocytic lymphohistiocytosis (HLH), and septic shock [3]. Neurological symptoms and complications are rare, with meningitis and encephalitis accounting for about 0.2% of cases [5]. Only two previously reported cases of HGA are associated with cerebral infarctions [6,7]. Given the recent increase in HGA cases, there may be more stroke presentations related to anaplasmosis that are currently being missed. By reporting a third case of HGA associated with stroke, the authors hope to raise awareness of this manifestation of anaplasmosis.

## Case presentation

A 65-year-old woman presented to the emergency department (ED) with abdominal pain, nausea, vomiting, and dizziness. Her past medical history was significant for uncontrolled diabetes, hypertension, chronic kidney disease (CKD) stage V, and a cerebrovascular accident in 2015 with residual right-sided weakness.

Her vital signs were within normal limits, and on examination, a systolic murmur and mild left-sided abdominal and flank tenderness were present. She was alert and oriented to name, place, and time. As seen in Table 1, her labs were remarkable for WBC 10.9 K/uL (4 - 10.4 K/uL), hemoglobin 10.9 g/dL (11.7 - 15.0 g/dL), and creatinine 4.5 mg/dL (0.60 - 1.00 mg/dL), which was increased from her baseline of 3.86 mg/dL (0.60 - 1.00 mg/dL). Chest X-ray showed hypoventilatory changes but no consolidations. CT abdomen and pelvis showed partially imaged bilateral lower lobe mosaic attenuation with differentials including infectious/inflammatory process but no biliary ductal dilation, pancreatic inflammation, or bowel obstruction. She developed a fever of 38.1 C, after a couple of hours in the ED. The imaging findings were thought to be consistent with early changes seen in community-acquired pneumonia and nausea and vomiting were attributed to acute kidney injury from dehydration. Hence, she was given intravenous fluids and discharged with a seven-day course of azithromycin.

**Figure 1:**
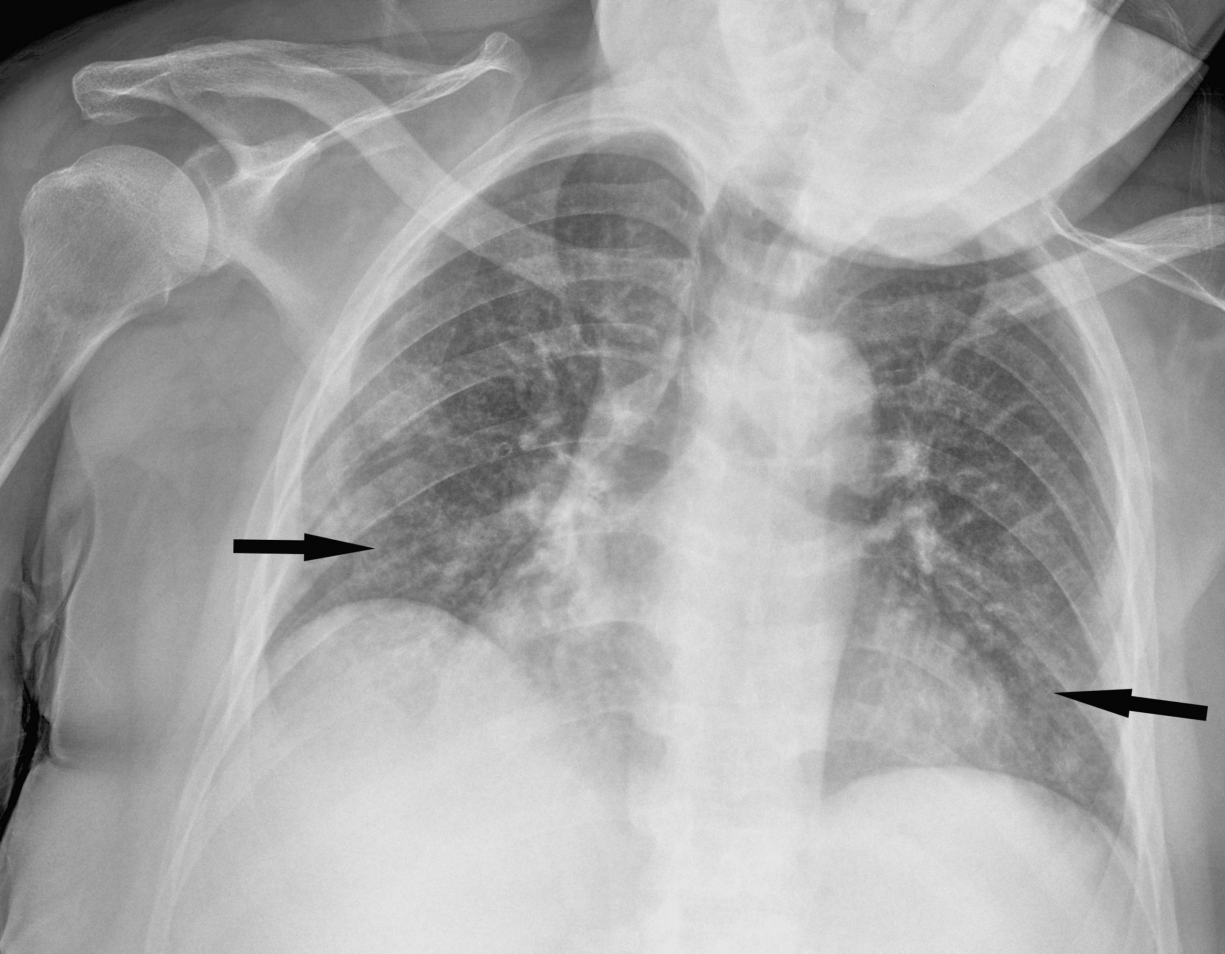
Chest X-ray with hazy opacities in the right mid, right lower (black arrow), and left lower lung (black arrow), consistent with multifocal pneumonia

She was at home for three days before returning to the ED for persistent symptoms of nausea, vomiting, abdominal pain, dizziness, and new-onset confusion. She was afebrile and hemodynamically stable but now only oriented to name; her physical exam was unremarkable. Her labs showed pancytopenia with WBC 3.2 K/uL (4.0 - 10.4 K/uL), hemoglobin 9.0 g/dL (11.7 - 15.0 g/dL), platelet 61 K/uL (150 - 350 K/uL), and creatinine 4.7 mg/dL (0.60 - 1.00 mg/dL) as seen in Table 1. On hospital day (HD) 1, chest X-ray (Figure 1) was consistent with multifocal pneumonia. After blood cultures were collected, she was started on vancomycin and cefepime. To evaluate her abdominal pain, abdominal ultrasound was done, which showed no biliary obstruction and labs showed aspartate aminotransferase (AST) 69 (0 - 32 unit/L), alanine aminotransferase (ALT) 42 (0-33 unit/L), alkaline phosphatase (ALP) 202 (35 - 115 unit/L), and total bilirubin 0.8 mg/dL (0.0 - 1.2 mg/dL).

**Table 1:** Comparing laboratory results from the initial ED visit to the second ED visit (Hospital Day 1) AST: aspartate aminotransferase; ALT: alanine aminotransferase; ALP: alkaline phosphatase; BUN: blood urea nitrogen

Labs	Initial ED visit	Second ED visit (Hospital Day 1)
WBC	10.9 K/uL (4.0 - 10.4 K/uL)	3.2 K/uL (4.0 - 10.4 K/uL)
Hemoglobin	10.9 g/dL (11.7 - 15.0 g/dL)	9.0 g/dL (11.7 - 15.0 g/dL)
Platelet	321 K/uL (150 - 350 K/uL)	61 K/uL (150 - 350 K/uL)
BUN	49 mg/dL (6 - 23 mg/dL)	51 mg/dL (6 - 23 mg/dL)
Creatinine	4.50 mg/dL (0.60 - 1.00 mg/dL)	4.78 mg/dL (0.60 - 1.00 mg/dL)
AST	26 unit/L (0 - 32 unit/L)	69 unit/L (0 - 32 unit/L)
ALT	16 unit/L (0 - 33 unit/L)	42 unit/L (0 - 33 unit/L)
Alkaline phosphatase	218 unit/L (35 - 115 unit/L)	202 unit/L (35 - 115 unit/L)
Total bilirubin	0.3 mg/dL (0.0 - 1.2 mg/dL)	0.8 mg/dL (0.0 - 1.2 mg/dL)

She became progressively more encephalopathic over the next few days; the leading thought process was likely worsening uremia. On HD 4, blood urea nitrogen (BUN) increased to 56 mg/dL (6 - 23 mg/dL) and creatinine increased to 5.57 mg/dL (0.60 - 1.00 mg/dL). Hemodialysis started through a newly placed tunneled dialysis catheter on HD 4. Her mentation continued to worsen. Hence, cefepime was switched to ceftriaxone on HD 5 with concern for possible cefepime-induced encephalopathy.

**Figure 2:**
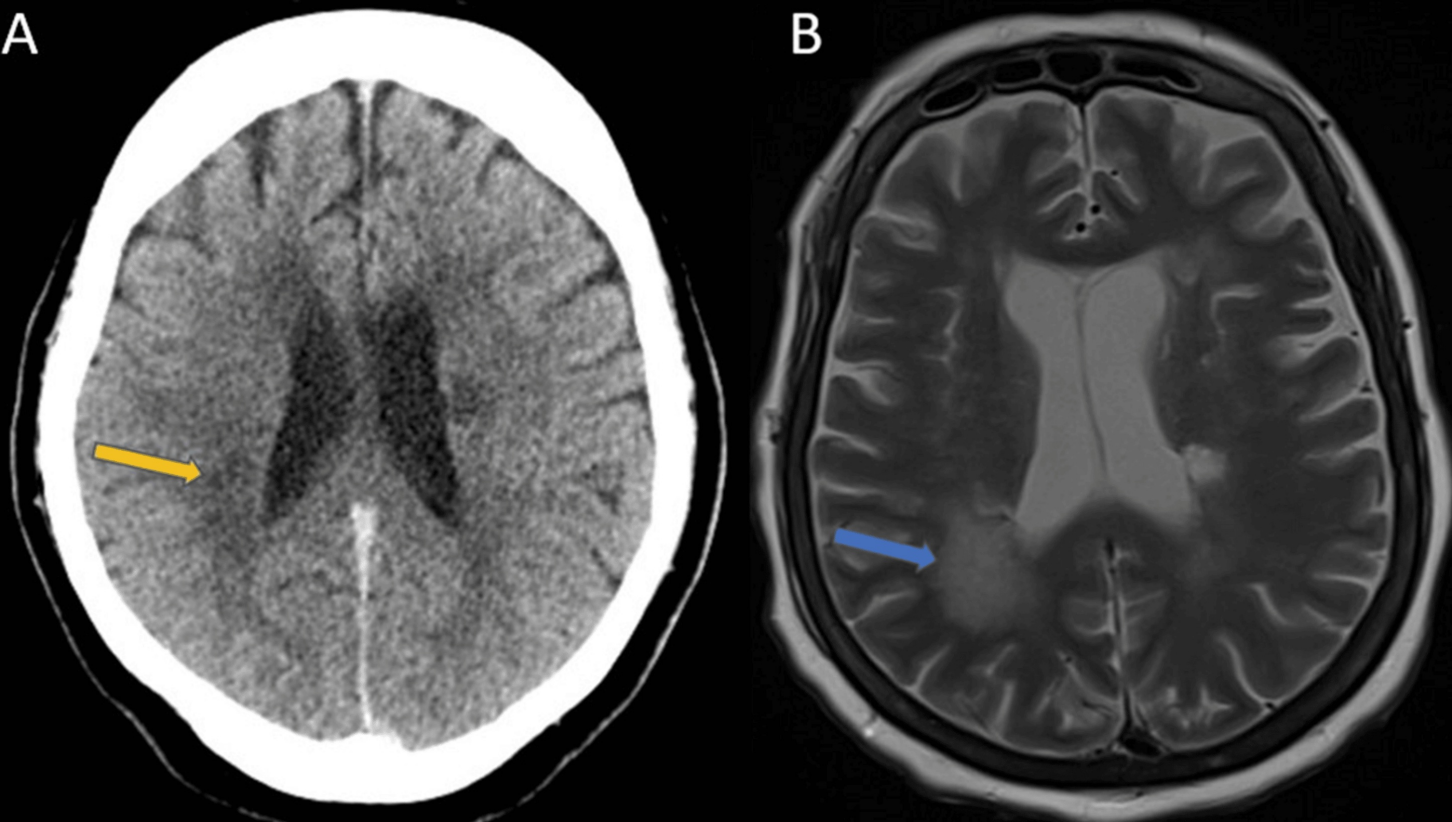
A. CT head without contrast. B. MRI brain without contrast Figure A. CT head without contrast showing a mildly increased component of ill-defined hypodensity in the posterior right corona radiata (orange arrow) consistent with subacute to acute infarction. Figure B. MRI brain without contrast showing a large, approximately 3 cm in maximal dimension area of the restricted diffusion between the subcortical white matter and the lateral wall of the posterior aspect of the body of the right lateral ventricle (blue arrow). Also, smaller areas of restricted diffusion (not seen on this slice of the image) are located at the watershed area. These findings are consistent with acute infarction possibly due to emboli etiology.

On HD 6, she had another fever with new leukocytosis; repeat blood cultures were sent, but the same antibiotics were continued. On HD 9, CT chest showed bilateral pulmonary edema. Infectious disease (ID) was consulted due to persistent fevers. On examination, she only responded to painful stimuli at this time. ID team recommended CT abdomen/pelvis to look for possible intraabdominal sources of infection and CT head without contrast to evaluate the etiology of worsening mentation. CT head without contrast showed a small area of mildly increased ill-defined hypodensity in the posterior right corona radiata (Figure 2A). CT abdomen/pelvis showed no acute abnormalities.

On HD 7, her fever curve had worsened, and she developed hemodynamic instability requiring vasopressors. She had progressive encephalopathy. On exam, she had a Glasgow Coma Scale (GCS) of 9, intermittently following simple commands but mostly grunting and withdrawing to pain. Hence, she was intubated for airway protection.

On HD 11, empiric meningitis coverage was started, ampicillin and acyclovir were added to vancomycin, and piperacillin-tazobactam was switched to ceftriaxone. Despite treatment, over the next 24 hours, fevers worsened with increasing vasopressor requirement. Given the lack of response to broad-spectrum antibiotics, with elevated liver enzymes and leukopenia in a patient during summertime, the team considered possible tick-borne infections and possible candidemia since a hemodialysis line was placed recently. Hence, on HD 12, repeat blood and sputum cultures were sent, after which doxycycline and micafungin were added to the regimen.

On the afternoon of HD 12, the EEG was negative and MRI brain showed multiple areas of restricted diffusion in the supratentorial brain, including a large, approximately 3 cm in maximal dimension area of restricted diffusion between the subcortical white matter and the lateral wall of the posterior aspect of the body of the right lateral ventricle. These findings were suggested of acute infarction, likely due to embolic etiology (Figure 2B).

**Figure 3:**
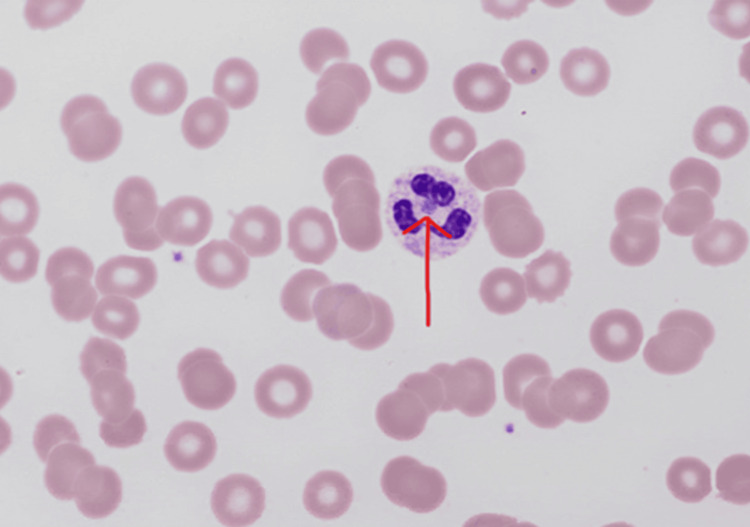
Neutrophilic inclusions are consistent with morulae (red arrow)

On HD 13, additional patient history was obtained from family members, noting that their home is surrounded by woods with deer in the backyard. The patient spent about two hours every day on the porch. The infectious disease team considered that the ticks were probably close enough to bite the patient on the porch. Hence, a peripheral blood parasite smear was sent, which showed rare neutrophil inclusions consistent with morulae highly suspicious for anaplasmosis (Figure 3).

On the afternoon of HD 13, a lumbar puncture was done, which showed white blood cell (7/uL), lymphocyte (78%), protein (28 mg/dL; range: 15-45 mg/dL), and glucose (136 mg/dL; range: 40-70 mg/dL), and other cerebrospinal fluid (CSF) studies were negative. These CSF findings were consistent with previously reported cases of HGA meningitis where they found slightly elevated WBCs with lymphocyte predominance with normal protein and glucose [8]. Hence, to confirm the diagnosis, a serum tick-borne PCR panel was sent, which was positive for *Anaplasma phagocytophilum*. The panel was negative for *Ehrlichia* species, *Babesia* species, and *Borrelia burgdorferi*. Also, the Lyme enzyme-linked immunosorbent assay (ELISA) screening serology was positive, with Western Blot positive for immunoglobulin (IgM) but negative for IgG, and the CSF antibody index was negative as seen in Table 2. The two possibilities were co-infection with early Lyme disease versus a false-positive IgM test. The team decided to extend doxycycline 100 mg twice daily for a total of 14 days to cover the CNS anaplasmosis and early Lyme disease co-infection.

**Table 2:** Results of the CSF and serum laboratory tests performed on the patient Ig: immunoglobulin; VDRL: Venereal Disease Research Laboratory

Test	Serum	CSF
*Anaplasma phagocytophilum *PCR	Detected	
*Babesia microti* PCR	Not detected	
*Ehrlichia chaffeensis* PCR	Not detected	
*Ehrlichia ewingii/canis* PCR	Not detected	
*Ehrlichia muris*-like PCR	Not detected	
*Borrelia burgdorferi* DNA PCR		Not detected
*Borrelia burgdorferi* ELISA screening IgM/IgG assay		
*Borrelia burgdorferi *Western Blot IgM	Positive	
*Borrelia burgdorferi* Western Blot IgG	Negative	
*Borrelia burgdorferi* antibody index		Negative
Powassan virus PCR		Negative
Treponemal Ab screen	Nonreactive	
VDRL		Negative
*Listeria monocytogenes* PCR		Not detected
*Escherichia coli* PCR		Not detected
*Neisseria meningitidis* PCR		Not detected
*Haemophilus influenzae* PCR		Not detected
*Streptococcus pneumoniae* PCR		Not detected
*Streptococcus agalactiae* PCR		Not detected
*Herpes simplex virus* 1 PCR		Not detected
*Herpes simplex virus* 2 PCR		Not detected
*Human Herpesvirus* 6 PCR		Not detected
*Varicella zoster virus* PCR		Not detected
*Enterovirus* PCR		Not detected
*Cytomegalovirus* PCR		Not detected
Cryptococcal antigen	Negative	
*Cryptococcus neoformans/gattii *PCR		Not detected

On HD 14, she was extubated and weaned off vasopressors. After two days on doxycycline, her mentation improved, and her fevers resolved. For the stroke, her aspirin was continued, levetiracetam was added for seizure prophylaxis, and later, atorvastatin for stroke prevention.

## Discussion

*Anaplasma phagocytophilum* is a small, gram-negative, obligate intracellular bacterium that is transmitted through the *Ixodes scapularis* (black-legged tick) in the eastern US region and *Ixodes pacificus* (western black-legged tick) in the western US region [9]. HGA was first reported in the United States in 1990, but at that time, it was called human granulocytic ehrlichiosis until it was switched to the *Anaplasma* genus and renamed in 2001 to HGA [10,11]. Large reservoirs of this bacteria are found in the white-tailed deer and white-footed mouse [12]. The nymphal stage of *Ixodes scapularis* ticks are smaller and, hence, are more likely to be attached to humans for long enough to transmit the infection compared to large adult ticks, which are easily identified and removed. Hence, the largest peak of HGA cases occurs between May and August, which corresponds with the greatest feeding activity of nymphal Ixodes ticks while a smaller second peak is seen in October and November, which corresponds to the feeding activity of adult Ixodes ticks [2,13].

Infections caused by *Anaplasma* are called HGA because, after a tick bite, the bacteria disseminate in the blood, bone marrow, and spleen but largely survive in the polymorphonuclear cells [14]. The bacteria, once inside the neutrophils, enter a vacuole altered by secreted bacterial components that, through many different mechanisms, evade autophagy and lysosome fusion to the vacuole, allowing the organism to replicate [14,15]. The bacteria are transmitted within 24 hours of the bite, and symptoms usually occur about one to two weeks after exposure. The most common symptoms include fevers, headache, myalgias, arthralgias, gastrointestinal symptoms, such as abdominal pain, nausea, vomiting, and diarrhea, and complications such as respiratory failure, acute renal failure, septic shock, and HLH [3,4,9].

Neurological symptoms and complications are rare, with meningitis and encephalitis accounting for about 0.2% of the cases [5]. There are rare cases of HGA causing bilateral facial palsy, demyelinating polyneuropathy, and brachial plexopathy previously reported [14]. Transient encephalopathy from HGA has been seen in elderly and immunocompromised patients [16]. There have been only two previously reported cases of HGA associated with infarctions and one case of subarachnoid hemorrhage [6,7,17].

In our patient, multiple foci with restricted diffusion in the supratentorial brain were seen as consistent with acute infarction with concern for an embolic etiology. There have been reported cases of cerebrovascular vasculitis with infarctions noted in humans with other tick-borne infections, such as Rickettsia, with autopsies of patients with Rocky Mountain spotted fever showing non-occlusive fibrin thrombi in various organs [18,19]. Mice infected with *Rickettsia conorii* showed procoagulant activity with a rise in factor V levels, decreased antithrombin concentrations, and plasma factor VIII activity [20]. There has been a previously reported hypothesis that anaplasmosis infects endothelial cells as well, which could contribute to stroke presentation [8]. Through more recognition of this presentation in patients with anaplasmosis, future studies could shed light on the mechanisms involved in causing strokes.

Laboratory findings were seen in HGA leukopenia, anemia, thrombocytopenia, and elevated liver enzymes, especially AST and acute kidney injury with elevated creatinine seen in nearly 25% of cases [3,9]. Per CDC, the diagnosis of HGA is based on confirmatory laboratory evidence and at least one of the objective or subjective clinical evidence criteria. The laboratory evidence consists of at least one of the following: *Anaplasma phagocytophilum *NAAT or PCR or other molecular testing, four-fold change in IgG Ab titer to* Anaplasma phagocytophilum* with one sample taken within two weeks after illness onset, and a second sample between 2 and 10 weeks after the first sample, or *Anaplasma* antigen in a biopsy or autopsy sample by immunohistochemical methods [21]. Though intracytoplasmic morulae in granulocytes can be seen in HGA, it is not a part of the laboratory confirmatory evidence. Only about 40% of PCR-positive patients have morulae seen on smear [21,22]. Clinical objective data includes anemia, leukopenia, thrombocytopenia, transaminase elevation, elevated CRP, and recorded fever while subjective clinical evidence includes headache, myalgia, chills, sweats, fatigue, or malaise [21].

The PCR test is the most sensitive (up to 100%) and specific test with higher sensitivity within 4 days of clinical illness onset but can detect DNA up to 30 days after illness onset compared to peripheral blood smear, which can detect morulae between 2 to 14 days of illness onset only. Serological response takes time to mount to the infection and can be negative in acute illness [21]. The requirement of a fourfold increase in titers with samples two weeks apart for diagnosis makes it less useful in acute diagnosis. Our patient had subjective clinical findings that were consistent, such as nausea, vomiting, and abdominal pain, and objective clinical data with leukopenia, anemia, thrombocytopenia, and elevated liver enzymes. In addition, the peripheral blood smear showed morulae, and a positive serum PCR test confirmed the diagnosis.

The treatment of HGA is oral doxycycline 100 mg twice daily for 7 to 10 days, but in patients with severe allergies or intolerance, oral rifampin 300 mg twice daily for 7 to 10 days can be used [16]. *Anaplasma* *phagocytophilum* is transmitted by the same *Ixodes scapularis* tick as *Borrelia burgdorferi*; hence, co-infections can be seen with about 13.1% co-infection reported in Pennsylvania [3]. This was seen in our patient who had Western Blot IgM positive suggesting possible co-infection with early Lyme disease. Also, laboratory testing for Lyme disease can be falsely negative earlier on in the disease process; hence, a longer duration of 10-14 days can be considered.

## Conclusions

There is an increase in HGA cases in the USA, particularly in Pennsylvania. We present the third case of stroke associated with HGA, but this phenomenon may be under-reported, given that the sensitivity of HGA serology and peripheral parasite smear is largely dependent on the timing of the tests. Newer tests that are more sensitive, such as *Anaplasma* PCR, could lead to more recognition of this presentation, and future studies could shed light on the mechanisms involved in causing strokes. We hope to highlight that though neurological manifestations are rare in HGA, if a patient from an endemic region has neurological symptoms during the summer or fall and has a history of tick bites or consistent laboratory abnormalities, HGA should be considered in the differential diagnosis.
